# Personal use of hair dyes and risk of leukemia: a systematic literature review and meta‐analysis

**DOI:** 10.1002/cam4.1162

**Published:** 2017-09-18

**Authors:** Kevin M. Towle, Matthew E. Grespin, Andrew D. Monnot

**Affiliations:** ^1^ Cardno ChemRisk 101 2nd St. Suite 700 San Francisco California 94105; ^2^ Cardno ChemRisk 4840 Pearl E. Cir Boulder Colorado 80301

**Keywords:** Epidemiology, hair dye, leukemia, meta‐analysis

## Abstract

The objective of this study was to examine the association between personal use of hair dyes and the risk of leukemia. We conducted a systematic literature review of epidemiology studies reporting leukemia‐specific cancer risks among hair dye users, and estimated the meta‐relative risk (meta‐RR) and corresponding 95% confidence interval (95% CI) of leukemia, comparing hair dye users to nonusers. When data from all 20 studies that met the inclusion criteria were combined, ever use of hair dye was associated with a nonstatistically significant increased risk of leukemia, meta‐RR = 1.09 (95% CI: 0.97–1.22). When restricted to studies that adjusted for smoking, ever use of hair dye was not associated with leukemia, meta‐RR = 0.99 (95% CI: 0.76–1.29). A statistically significant increased risk of leukemia was associated with permanent hair dye use (meta‐RR = 1.19 [95% CI: 1.07–1.33]), dark hair dye use (meta‐RR = 1.29 [95% CI: 1.11–1.50]), hair dye use among males (meta‐RR = 1.42 [95% CI: 1.01–2.00]), hair dye use pre‐1980 (meta‐RR = 1.49 [95% CI: 1.21–1.83]), and hair dye use for ≥15 years (meta‐RR = 1.35 [95% CI: 1.13–1.62]). Overall, findings suggest that ever use of hair dye is not a significant risk factor for leukemia. Certain hair dye use characteristics were associated with a statistically significant increased risk, but further research is required to determine whether these associations truly reflect a risk of leukemia due to methodological limitations in the underlying studies.

## Introduction

The prevalence of the use of hair dye, a mixture of chemical compounds used to treat hair for cosmetic purposes, has increased worldwide, with an estimated 50–80 percent of women in the United States, Japan, and European Union reporting to have used hair dye in their lifetime 1. Globally, the cosmetic manufacturing industry reported $255 billion in revenue in 2014, with hair care products accounting for nearly a quarter of industry revenue 2.

Hair dye products include a range of over 5000 chemical compounds and are composed of dye intermediates and dye couplers that interact with each other in the presence of hydrogen peroxide to form pigment molecules 3, 4. Aromatic amines, such as para‐phenylenediamine, are present in many hair dye products in order to impart hair color and prevent fading due to washing 5, 6. At certain doses, some aromatic amines have been shown to be carcinogenic in animals and to significantly induce cytotoxic and genotoxic effects in human cells 6, 7, 8, 9, 10. The primary carcinogenic mechanism of action is believed to involve CYP‐450‐mediated oxidation of parent aromatic amines to a carcinogenic metabolite 5. It seems reasonable to assume that metabolic activation and subsequent genotoxic effects could occur in any tissue where reactive metabolites of aromatic amines accumulate to a significant degree, yet to date, bladder cancer appears to be the only cancer endpoint that is consistently increased in humans exposed to elevated levels of these compounds 11, 12, 13. The International Agency for Research on Cancer (IARC) recently concluded that aromatic amine dyes are “probably carcinogenic” in hairdressers and barbers, based on “limited evidence” of an increased bladder cancer risk in the underlying epidemiology studies 1.

The association between personal use of hair dye (involving self‐application) and cancer endpoints has also been evaluated, and inconsistent findings have been reported that vary by cancer type 14, 15, 16. Takkouche et al. conducted a meta‐analysis of the available data in 2005 and concluded that there was no “strong evidence of a marked increase in the risk of cancer among personal hair dye users,” but observed a “borderline” increased risk of hematopoietic cancers in the aggregated results of 40 studies (RR = 1.15; 95% CI, 1.05–1.27) 17. Lymphocytic leukemia had one of the highest relative risks of all the hematopoietic disease endpoints evaluated (RR = 1.41; 95% CI, 1.06–1.88). More recently, IARC concluded that studies of personal hair dye users yielded inconclusive results and that personal hair dye use was therefore “not classifiable as to its carcinogenicity to humans” 1.

Leukemia, a type of hematopoietic cancer that arises in the bone marrow, is classified by the rate of growth (acute or chronic) and cell line of origin (lymphocytic or myeloid). According to the Surveillance, Epidemiology, and End Results Program (SEER), rates for new leukemia cases have been rising on average 0.3 percent per year over the last 10 years, with over 60,000 new cases of leukemia projected to occur in 2016 18. To our knowledge, aromatic amines have not been shown to be a cause of leukemia in animal studies or in occupational settings, yet as noted in the Takkouche analysis of personal hair dye users, “some aspects related to hematopoietic cancer should be investigated further” 17.

Since the meta‐analysis of Takkouche in 2005, ten studies have been published in which the association between personal hair dye use and the risk of one or more leukemia subtypes was evaluated 3, 19, 20, 21, 22, 23, 24, 25, 26, 27, 28. Many of these more recent studies provided more robust information on gender and hair dye use patterns, including hair dye type and time period/duration of use. The goal of this study was to perform a current meta‐analysis of all peer‐reviewed epidemiology studies that have reported leukemia‐specific cancer risks among personal hair dye users. Stratified analyses were performed to assess risk factors.

## Material and Methods

### Literature searches

We followed standardized PRISMA protocol and performed a systematic literature review to estimate the pooled relative risk of leukemia among personal hair dye users. Studies were identified by electronic database searching of PubMed, Web of Science, and Embase from their respective inception dates, using the search terms “hair” and (“dye” or “color” or “colour”) and (“leukemia” or “leukaemia” or “myeloid” or “lymphocytic” or “myelogenous” or “lymphoblastic”). Findings were supplemented with references manually obtained from the search results. Furthermore, we systematically reviewed studies that cited the articles that met our inclusion criteria. The latest search was conducted in May 2017.

### Study selection

**Figure 1:**
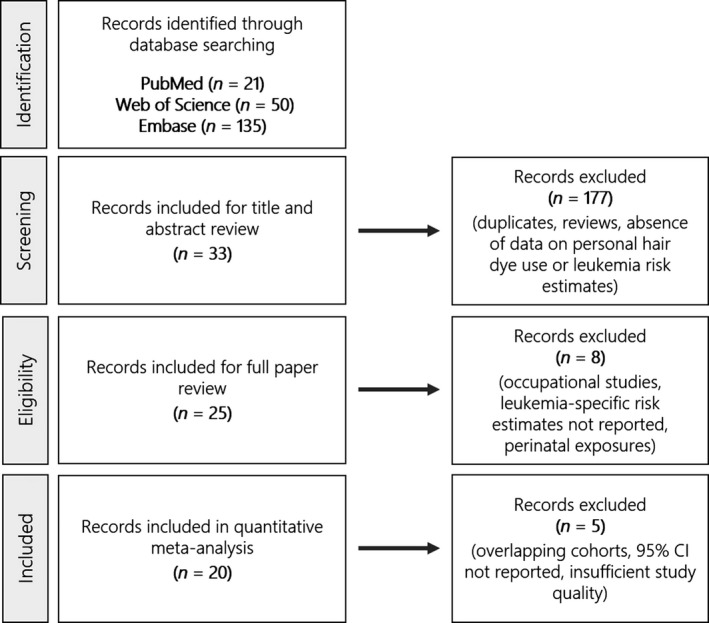
Flow chart of literature review and paper selection following inclusion and exclusion criteria.

Two independent individuals reviewed and screened the title and abstract results to identify potentially relevant articles (Fig. 1). Selection criteria for full article review included (1) data on personal hair dye use, (2) all types of leukemia or subtypes of leukemia studied, (3) cohort or case–control studies, (4) reported risk estimates and 95% confidence intervals (95% CI), or available data for the calculation of measures of association. We excluded occupational studies examining hairdressers, preleukemia health endpoints (i.e., essential thrombocythemia and myelodysplastic syndromes), perinatal exposure to hair dye use, reviews, editorials, and articles not written in English. Additionally, studies reporting pooled risk estimates for both leukemia and general lymphoma endpoints grouped together were excluded, but studies reporting risk estimates for specifically the chronic lymphocytic leukemia/small lymphocytic lymphoma (CLL/SLL) endpoint were included due to the similarity of diseases. For studies examining the same cohort, preference was given to the more comprehensive analysis with longer length of follow‐up or greater number of cases. The Kappa statistic was used to assess inter‐rater agreement, and any disagreements were resolved by consensus with all authors.

### Data abstraction

Data on study design, study population, primary exposure, subexposure, health outcome, risk estimates, confidence intervals, country of study, time period of diagnoses, time period of hair dye use data, number of cases and controls, cohort size, participation rate, and adjusted covariates were collected for each article. Study‐specific data were abstracted by one reviewer and assessed for quality control by an independent reviewer.

### Data synthesis and analysis

Each study was critiqued and analyzed for validity of study design and completeness of information based on potential for confounding, participation rate, loss to follow‐up, methodology of interviews or questionnaires, and selection protocol. Sensitivity analyses were performed to determine the effect of study exclusion.

Fixed‐effect and random‐effects (in the presence of heterogeneity [*P* < 0.05]) models were employed to estimate the pooled relative risk (meta‐RR) of all types of leukemia among all hair dye type users with 95% CI, weighted by the inverse of the study variance. Stratified analyses were performed by leukemia group (lymphocytic and myeloid), leukemia subtype (AML [acute myeloid leukemia and acute nonlymphocytic leukemia], CLL [chronic lymphocytic leukemia and small lymphocytic lymphoma], and ALL [acute lymphocytic leukemia and lymphoblastic leukemia]), duration of hair dye use (≤10 years or ≥15 years), time period of hair dye use (pre‐1980 and post‐1980), dark hair dye (black, brown, brunette, or classified as dark in paper), light hair dye (blonde or classified as light in paper), use of permanent hair dye, and gender. In these analyses, gender‐, type‐, and time period‐specific risk estimates were included in the models when studies reported separate measures of association by males and females, permanent and semi‐permanent hair dyes, and decade of hair dye use. No hair dye use was used as the reference group, and a minimum of four studies were required per stratified analysis. Cochran's *Q* test was used to examine study heterogeneity. Funnel plots (log relative risk and standard error) and Egger's test were performed to assess the potential of publication bias. All statistical analyses were performed using Stata software, version 14.2.

## Results

A total of 33 papers met the inclusion criteria for full paper review from the original 206 abstracts (Fig. 1). The Kappa statistic for inter‐rater agreement was 0.84, which is indicative of very good agreement. Twenty‐five papers underwent full data abstraction (Table 1), including 20 case–control and five cohort studies. Reviewed articles were published between 1985 and 2016, and contained information from various North American, European, and Asian countries, including Sweden (*n* = 2), Serbia (*n* = 1), China (*n* = 3), Denmark (*n* = 1), Italy (*n* = 4), Greece (*n* = 1), Spain (*n* = 1), United States (*n* = 7), and multiple countries (*n* = 5). The studies examined all leukemia types grouped together, as well as various subtypes of lymphocytic and myeloid leukemia.

**Table 1:** Abstracted data from selected studies

Author	Year	Study Design	Total *N* (exposed and unexposed)	*N* (exposed cases)	Exposure	Outcome	Risk Estimate	Lower 95% CI	Upper 95% CI	Time Period of Diagnosis	Study Population	Adjustment
Albin et al. 51	2000	Case‐control	333 cases; 351 controls	65	Ever	AML	0.98	0.64	1.5	1976–1993	Adults	Age, gender, and county
Altekruse et al. 30	1999	Cohort	547,586	207	Ever	All leukemias	1.1	0.9	1.3	1982–1994	Women	Age, race, smoking, education, occupation, and self‐reported exposure to X‐ray or fluoroscopic examinations, radium, radioactive isotopes, coal tar/asphalt, diesel exhaust, dyes, gasoline exhaust, pesticides, textiles, and dusts
			453,302	69	1–9 years	All leukemias	0.9	0.7	1.2			
			432,903	78	10–19 years	All leukemias	1.2	0.9	1.5			
			409,085	60	20 + years	All leukemias	1.3	1	1.7			
Benavente et al. 19	2005	Case–control	577 cases; 616 controls	37	Ever	CLL	2.3	1.1	4.7	1998–2002	Adults	Pathology center, gender, age, and house ownership
			113 cases; 520 controls	27	Before 1980	CLL	3.5	1.5	7.8			
			96 cases; 525 controls	10	After 1980	CLL	1.5	0.6	3.6			
			110 cases; 546 controls	24	Dark	CLL	2.3	1.1	4.9			
			103 cases; 510 controls	17	Light	CLL	2.9	1.3	6.8			
			121 cases; 589 controls	35	Permanent	CLL	3.4	1.4	7.8			
			89 cases; 456 controls	3	Semi‐permanent	CLL	1.6	0.4	6.6			
			92 cases; 502 controls	6	≤10 years	CLL	1.1	0.4	3			
			98 cases; 481 controls	12	11–24 years	CLL	3.2	1.3	8.2			
			105 cases; 491 controls	19	≥25 years	CLL	3.7	1.5	8.9			
Bjork et al. 34	2001	Case‐control	226 cases; 251 controls	25	Ever	CML	0.35	0.18	0.68	1976–1993	Adults	Gender, age, and county
Cantor et al. 52	1988	Case‐control	577 cases; 1245 controls	43	Ever	All leukemias	1.8	1.1	2.7	1980–1983	Men	State of residence, and age
			143 cases; 1245 controls	7	Ever	ANL	1.1	0.5	2.6			
			243 cases; 1245 controls	14	Ever	CLL	1.4	0.7	2.6			
			51 cases; 1245 controls	5	Ever	CML	2.2	0.7	6.2			
			16 cases; 1245 controls	2	Ever	ALL	2.9	0.4	13.8			
de Sanjose et al. 3	2006	Case‐control	407 cases; 2417 controls	127	Ever	CLL	1.43	1.01	2.03	1998–2003	Adults	Center, gender, educational level, home ownership, and age
Grodstein et al. 53	1994	Cohort	99,067	8	Ever	CLL	0.6	0.3	1.5	1976–1990	Women	Age
			99,067	8	Ever	AML/CML/ALL	0.8	0.3	1.9			
Karakosta et al. 20	2015	Case–control	171 cases; 175 controls	33	Ever	CLL	0.99	0.57	1.71	NR	Adults	Age, gender, ethnicity, area of residence, and family history of malignancy
Markovic‐Denic et al. 50	1995	Case‐control	130 cases; 130 controls	11	Ever	CLL	1.97	1.08	3.59	1989	Adults	Gender, age, place of residence, area of residence, and income
Markowitz et al. 33	1985	Case‐control	101 cases; 101 controls	NR	Ever	ANL	3.1	NR	NR	1980–1982	Adults	Matched (unspecified)
Mele et al. 31	1994	Case‐control	252 cases; 1161 controls	NR	Ever	AML	1.2	0.4	4	1987–1990	Men	Age, education, and residence
			156 cases; 1161 controls	NR	Ever	CML	2.1	0.7	6.2			
			252 cases; 1161 controls	NR	Dark	AML	1.6	0.4	5.5			
			156 cases; 1161 controls	NR	Light	CML	2	0.2	28.1			
			156 cases; 1161 controls	NR	Dark	CML	2.1	0.6	7.2			
			252 cases; 1161 controls	NR	Ever	AML	1	0.7	1.3		Women	
			100 cases; 1161 controls	NR	Ever	ALL	1.2	0.8	1.8			
			156 cases; 1161 controls	NR	Ever	CML	1	0.6	1.5			
			252 cases; 1161 controls	NR	Light	AML	0.7	0.4	1.2			
			252 cases; 1161 controls	NR	Dark	AML	1.2	0.7	2			
			100 cases; 1161 controls	NR	Light	ALL	1.4	0.6	3.1			
			100 cases; 1161 controls	NR	Dark	ALL	1.2	0.6	2.5			
			156 cases; 1161 controls	NR	Light	CML	1	0.4	2.1			
			156 cases; 1161 controls	NR	Dark	CML	1.1	0.6	2.2			
			252 cases; 1161 controls	NR	≤10 years	AML	0.7	0.4	1.2			
			252 cases; 1161 controls	NR	>10 years	AML	1.6	0.8	3			
			100 cases; 1161 controls	NR	≤10 years	ALL	1.2	0.6	2.3			
			100 cases; 1161 controls	NR	>10 years	ALL	2	0.7	5.7			
			156 cases; 1161 controls	NR	≤10 years	CML	0.9	0.5	1.8			
			156 cases; 1161 controls	NR	>10 years	CML	0.8	0.3	2.2			
Mele et al. 32	1995	Case‐control	36 cases; 1161 controls	NR	Ever	APL	1.5	0.6	3.7	1986–1990	Adults	Age, gender, education, and residence
			216 cases; 1161 controls	NR	Ever	Other AML	0.8	0.5	1.3			
Mendelsohn et al. 21	2009	Cohort	29	9	Ever	Leukemia	0.68	0.31	1.51	1996–2000	Women	Age, education, and smoking
			22	2	1–2 years	Leukemia	0.31	0.07	1.31			
			24	4	3–4 years	Leukemia	1.05	0.36	3.12			
			22	2	5–9 years	Leukemia	1.05	0.24	4.56			
			21	1	≥10 years	Leukemia	0.89	0.12	6.76			
Miligi et al. 54	1999	Case‐control	NR	NR	Ever	Leukemia	0.9	0.7	1.3	NR	Adults	Age
			NR	NR	Permanent	Leukemia	1.2	0.9	1.7			
			NR	NR	Dark	Leukemia	2	1.1	3.8			
Miligi et al. 22	2005	Case‐control	NR	6	Ever	Leukemia	0.6	0.3	1.6	NR	Men	Age and smoking
			NR	4	Permanent	Leukemia	1.2	0.3	4.1			
			NR	3	Brown	Leukemia	1.9	0.4	10.4			
			NR	140	Ever	Leukemia	1	0.7	1.3		Women	
			NR	114	Permanent	Leukemia	1.2	0.9	1.6			
			NR	20	Black	Leukemia	1.9	1	3.4			
			NR	39	Brown	Leukemia	1.2	0.7	1.8			
			NR	10	Red	Leukemia	1	0.4	2.1			
			NR	85	Permanent	All (excluding CLL)	1.1	0.8	1.6			
			NR	38	Non‐permanent	All (excluding CLL)	0.9	0.6	1.4			
			NR	37	Permanent	CLL	1.5	0.9	2.4			
			NR	12	Non‐permanent	CLL	0.9	0.4	1.8			
			NR	43	Blonde	Leukemia	0.9	0.6	1.3			
			NR	41	Permanent	Lymphocytic	1.3	0.8	2.2			
			NR	14	Non‐permanent	Lymphocytic	0.9	0.4	1.6			
			NR	58	Permanent	Myelocytic	1.1	0.7	1.6			
			NR	24	Non‐permanent	Myelocytic	0.8	0.5	1.4			
Rauscher et al. 55	2004	Case‐control	769 cases; 623 controls	185	Ever	Leukemia	1.3	0.99	1.8	1986–1989	Adults	Age, race, gender, geographic region, and education
			596 cases; 509 controls	12	Temporary	Leukemia	0.93	0.41	2.1			
			630 cases; 537 controls	47	Semi‐permanent	Leukemia	1.1	0.7	1.7			
			671 cases; 546 controls	104	Permanent	Leukemia	1.6	1.1	2.4			
			109 cases		Permanent	Lymphoblastic	2	0.87	4.6			
			515 cases		Permanent	Myelocytic	1.6	1.1	2.5			
			601 cases; 508 controls	17	1–4 years (permanent)	Leukemia	1.6	0.6	2.9			
			600 cases; 511 controls	16	1–4 years (semi‐permanent)	Leukemia	1	0.48	2.1			
			613 cases; 515 controls	29	5–14 years (permanent)	Leukemia	1.5	0.78	2.7			
			608 cases; 514 controls	24	5–14 years (semi‐permanent)	Leukemia	1.3	0.67	2.5			
			623 cases; 516 controls	39	≥15 years (permanent)	Leukemia	1.9	1.1	3.6			
			605 cases; 516 controls	21	≥15 years (semi‐permanent)	Leukemia	1	0.52	2			
			604 cases; 522 controls	50	Light (permanent)	Leukemia	1.8	1.1	3.1			
			609 cases; 511 controls	25	Dark (permanent)	Leukemia	1.6	0.78	3.2			
			606 cases; 522 controls	22	Light (semi‐permanent)	Leukemia	0.79	0.42	1.5			
			612 cases; 519 controls	28	Dark (semi‐permanent)	Leukemia	1.2	0.65	2.1			
			629 cases; 522 controls	45	Before 1970 (permanent)	Leukemia	1.7	0.98	3			
			611 cases; 512 controls	27	1970–1979 (permanent)	Leukemia	1.6	0.83	3.1			
			597 cases; 506 controls	13	After 1979 (permanent)	Leukemia	1.2	0.51	2.9			
			621 cases; 519 controls	37	Before 1970 (semi‐permanent)	Leukemia	1.6	0.87	2.8			
			603 cases; 515 controls	19	1970–1979 (semi‐permanent)	Leukemia	0.98	0.5	1.9			
			589 cases; 508 controls	5	After 1979 (semi‐permanent)	Leukemia	0.41	0.14	1.2			
Sandler et al. 56	1993	Case‐control	615 cases; 630 controls	NR	Ever	Leukemia	1.5	1.1	2.1	NR	Adults	Age, race, gender, smoking, and income
			NR	NR	Permanent	Leukemia	1.6	1	2.4			
			NR	NR	Semi‐permanent	Leukemia	1.4	0.9	2.1			
			NR	NR	Temporary	Leukemia	1.2	0.3	4			
			NR	NR	≥16 years	Leukemia	2.6	NR	NR			
Skibola et al. 23	2014	Case‐control	38 cases; 4680 controls	27	Ever	ALL	1	0.48	2.11	NR	Adults	Age and gender
			13 cases; 2100 controls	2	Temporary	ALL	2	0.36	11.02			
			35 cases; 4245 controls	24	Permanent	ALL	0.94	0.44	2.03			
			22 cases; 2654 controls	11	Light	ALL	1.39	0.57	3.34			
			24 cases; 3523 controls	13	Dark	ALL	0.82	0.35	1.89			
			23 cases; 2650 controls	12	1–8 years	ALL	0.83	0.34	2.02
			19 cases; 2413 controls	8	9–19 years	ALL	1.06	0.40	2.80
			14 cases; 2491 controls	3	20+ years	ALL	0.7	0.18	2.71
			16 cases; 2863 controls	5	Before 1980	ALL	1.07	0.33	3.5
			30 cases; 2702 controls	19	After 1980	ALL	1.23	0.5	3
Slager et al. 24	2014	Case‐control	404 cases; 4122 controls	284	Ever	CLL/SLL	1.08	0.86	1.37	Until 2011	Adults	Age, gender, race, and study
			134 cases; 1447 controls	14	Temporary	CLL/SLL	0.74	0.41	1.34			
			379 cases; 3621 controls	259	Permanent	CLL/SLL	1.16	0.91	1.48			
			218 cases; 2135 controls	98	Light	CLL/SLL	1.13	0.84	1.51			
			292 cases; 2918 controls	172	Dark	CLL/SLL	1.11	0.86	1.44			
			192 cases; 2162 controls	72	1–8 years	CLL/SLL	1	0.71	1.42			
			191 cases; 1918 controls	71	9–19 years	CLL/SLL	1.22	0.86	1.72			
			215 cases; 2011 controls	95	≥20 years	CLL/SLL	1.26	0.92	1.73			
			247 cases; 2136 controls	127	Before 1980	CLL/SLL	1.36	1	1.86			
			216 cases; 2231 controls	96	After 1980	CLL/SLL	1.06	0.76	1.46			
Thun et al. 29	1994	Cohort	85	16	Ever	Lymphoid	0.77	0.44	1.35	1982–1989	Women	Age
			74	5	1–9 years	Lymphoid	0.71	0.27	1.86			
			76	7	10–19 years	Lymphoid	0.91	0.4	2.08			
			73	4	≥20 years	Lymphoid	0.67	0.24	1.86			
			198	49	Ever	Myeloid and Monocytic	0.93	0.67	1.3			
			169	20	1–9 years	Myeloid and Monocytic	1.02	0.61	1.69			
			168	19	10–19 years	Myeloid and Monocytic	0.97	0.6	1.58			
			159	10	≥20 years	Myeloid and Monocytic	0.79	0.42	1.51			
			82	18	Ever	Other	1.01	0.57	1.79			
			73	9	1–9 years	Other	1.66	0.76	3.61			
			71	7	10–19 years	Other	0.87	0.38	2.02			
			66	2	≥20 years	Other	0.47	0.11	2.08			
Vedel‐Krogh et al. 25	2016	Cohort	45	5	Ever	Leukaemia	0.40	0.16	1.02	1976–1978	Women	Age, birth year, BMI, smoking, alcohol, physical activity, marital status, education, income, systolic blood pressure, cholesterol, triglycerides, glucose, FEV1, and FEV1/FVC
Wong et al. 27	2009	Case‐control	263 cases; 530 controls	NR	Ever	AML	0.98	0.8	1.2	2003–2007	Adults	Age and gender
Wong et al. 28	2010	Case‐control	15 cases; 56 controls	NR	Ever	CLL/SLL	0.37	0.18	0.76	2003–2008	Adults	Age and gender
Zahm et al. 57	1992	Case‐control	37 cases; 723 controls	3	Ever	CLL	1	0.2	3.8	1983–1986	Men	Age
			35 cases; 682 controls	1	Semi or non‐permanent	CLL	3.2	0.1	28.6			
			19 cases; 695 controls	9	Ever	CLL	1	0.3	2.6		Women	
			17 cases; 623 controls	7	Semi or non‐permanent	CLL	0.9	0.3	2.8			
			12 cases; 482 controls	2	Permanent	CLL	0.8	0.1	4			
			11 cases; 414 controls	1	Blonde (semi‐ or nonpermanent)	CLL	1.1	0.05	9.1			
			14 cases; 511 controls	4	Brown/brunette (semi‐ or nonpermanent)	CLL	1	0.3	3.6			
			12 cases; 416 controls	2	Brown/brunette (permanent)	CLL	1.8	0.3	9.9			
Zhang et al. 26	2008	Case‐control	324 cases	244	Ever	CLL/SLL	1.3	1	1.6	1988–2003	Women	Age, race, and study center
			243 cases	163	Permanent	CLL/SLL	1.2	0.9	1.6			
			181 cases	101	Nonpermanent	CLL/SLL	1.3	0.9	1.7			
			228 cases	148	Dark	CLL/SLL	1.2	0.9	1.7			
			184 cases	104	Light	CLL/SLL	1.3	0.9	1.8			
			239 cases	159	Before 1980	CLL/SLL	1.5	1.1	2			
			164 cases	84	After 1980	CLL/SLL	1.3	1	1.7			
			NR	NR	≥20 years	CLL/SLL	1.3	1	1.8			

AML, acute myeloid leukemia; ALL, acute lymphocytic leukemia; CML, chronic myeloid leukemia; CLL, chronic lymphocytic leukemia; CLL/SLL, chronic lymphocytic leukemia/small lymphocytic lymphoma; NR, not reported.

Due to the presence of overlapping cohorts, Thun 1994 and Zhang 2008 were removed from the analyses, with preference given to the more comprehensive analyses of Altekruse 1999 and Slager 2014, respectively 24, 26, 29, 30. Additionally, Mele 1995 was excluded, as it analyzed a subset of patients from the Mele 1994 study 31, 32. Markowitz 1985 did not report 95% CI, and was therefore also excluded from this analysis 33. Based on data completeness review, Bjork 2001 was excluded from the analysis due to crude exposure classifications, no adjustment for smoking, and hair dye use information obtained by next of kin interviews in 81 percent of cases and only 14 percent of controls, which could introduce bias to the calculated odds ratio 34. Sensitivity analyses indicated that the conclusions did not significantly change when Bjork was included in the model.

**Figure 2:**
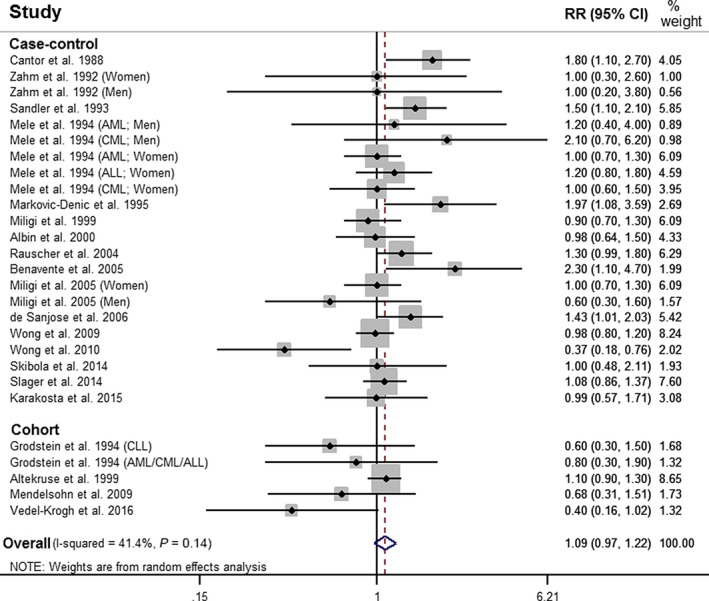
Forest plot of risk of leukemia, meta‐RR 1.09 (95% CI: 0.97–1.22), comparing ever use of personal hair dye to no use of personal hair dye. RR, relative risk; CI, confidence interval; AML, acute myeloid leukemia; CML, chronic myeloid leukemia; ALL, acute lymphocytic leukemia; CLL, chronic lymphocytic leukemia.

Based on 20 studies, ever use of any type of personal hair dye was associated with a nonstatistically significant increased risk of leukemia, when compared to no use of hair dye, meta‐RR = 1.09 (95% CI: 0.97–1.22) (Fig. 2). A model restricted to case–control studies yielded a statistically significant increased meta‐RR of 1.13 (95% CI: 1.00–1.28), while a model including cohort studies yielded a meta‐RR of 1.00 (95% CI: 0.85–1.19) (Table 2). When restricted to studies that adjusted for smoking history, use of any hair dye was not associated with leukemia, meta‐RR = 0.99 (95% CI: 0.76–1.29).

**Table 2:** Meta‐RR for leukemia among all models, comparing hair dye users to nonusers

	Number of studies (*n*)	Meta‐RR	95% CI	Heterogeneity *P*‐value	*I* ^2^ (%)
Any dye use	20	1.09	0.97–1.22	0.014	41.4
Any dye use (case–control)	16	1.13	1.00–1.28	0.024	41.2
Any dye use (cohort)	4	1.00	0.85–1.19	0.113	46.5
Any dye use (smoking adjusted)	5	0.99	0.76–1.29	0.037	57.9
Dark hair dye	8	1.29	1.11–1.50	0.804	0
Light hair dye	7	1.13	0.95–1.34	0.190	26.7
Permanent hair dye	10	1.19	1.07–1.33	0.207	24.1
Semi or non–permanent hair dye	7	1.05	0.86–1.29	0.854	0
Males	4	1.42	1.01–2.00	0.204	32.6
Females	7	1.02	0.90–1.15	0.517	0
Pre–1980	4	1.49	1.21–1.83	0.361	8.9
Post‐1980	4	1.06	0.81–1.38	0.439	0
Lymphocytic Leukemia	13	1.17	1.03–1.34	0.054	39.2
Myeloid Leukemia	6	1.06	0.93–1.20	0.511	0
Acute Myeloid Leukemia	4	0.99	0.85–1.16	0.989	0
Chronic Lymphocytic Leukemia	10	1.15	0.90–1.46	0.020	51.4
Acute Lymphocytic Leukemia	4	1.29	0.93–1.78	0.485	0
≥15 years of use	5	1.35	1.13–1.62	0.149	38.5
≤10 years of use	7	0.94	0.80–1.11	0.869	0

When stratified by subtype of leukemia, use of hair dye was associated with a statistically significant increased risk of lymphocytic leukemia (CLL, ALL, CLL/SLL, and lymphocytic endpoints), meta‐RR = 1.17 (95% CI: 1.03–1.34), and a meta‐RR = 1.06 (95% CI: 0.93–1.20) for myeloid leukemia (AML, CML, and myelocytic endpoints). No statistically significant associations were observed when models were separated by AML, CLL, and ALL subtypes individually (Table 2). Hair dye use among males was associated with a statistically significant increased risk of leukemia, meta‐RR = 1.42 (95% CI: 1.01–2.00), while hair dye use among females was not, meta‐RR = 1.02 (95% CI: 0.90–1.15).

**Figure 3:**
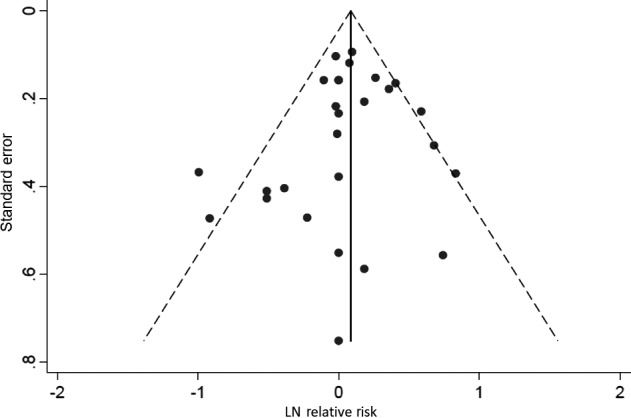
Funnel plot for all included studies (log risk estimate and standard error). Egger's test did not provide evidence of asymmetry (*P* = 0.53).

Use of dark hair dye and permanent hair dye were both associated with a statistically significant increased risk of leukemia, meta‐RR = 1.29 (95% CI: 1.11–1.50), and meta‐RR = 1.19 (95% CI: 1.07–1.33), respectively, while the use of light hair dye, meta‐RR = 1.13 (95% CI: 0.95–1.34), and semi‐permanent or nonpermanent hair dye, meta‐RR = 1.05 (95% CI: 0.86–1.29), were not. Additionally, use of hair dye prior to 1980 was associated with a statistically significant increased risk of leukemia, meta‐RR = 1.49 (95% CI: 1.21–1.83), while use of hair dye after 1980 was not, meta‐RR = 1.06 (95% CI: 0.81–1.38). Finally, use of hair dye for ≥15 years was associated with a statistically significant increased risk of leukemia, meta‐RR = 1.35 (95% CI: 1.13–1.62), while use of hair dye for ≤10 years was associated with a meta‐RR of 0.94 (95% CI: 0.80–1.11). A funnel plot and Egger's test did not show evidence of asymmetry (*P* = 0.53), and did not indicate the presence of publication bias (Fig. 3).

## Discussion

### Overview of findings

This analysis evaluated 25 studies published between 1985 and 2016 that reported leukemia‐specific risk estimates among hair dye users. The majority of selected studies reported relative risks by type of hair dye, duration/time period of use, gender, or leukemia subtype. A total of 149 individual risk estimates were considered in this analysis, 17 of which indicated a statistically significant increased risk of leukemia. When data from all studies were combined, we found that ever use of hair dye was associated with a nonstatistically significant increased risk of leukemia when compared to no use of hair dye. Findings also suggest that certain exposure characteristics, such as hair dye type (i.e., dark/permanent dye), time period of use (i.e., pre‐1980), duration of use (i.e., ≥15 years), and male gender, are associated with a statistically significant increased risk of leukemia. However, the observed findings may be due to the introduction of bias and confounding by smoking, study design limitations, and historical formulations of hair dye products.

### Risk by any hair dye

An unstratified meta‐analysis indicated that use of any type of hair dye was associated with a nonstatistically significant increased risk of all types of leukemia, meta‐RR 1.09 (95% CI: 0.97–1.22) (Table 2). This finding is in agreement with Takkouche 17 that reported a pooled RR of 1.12 (95% CI: 0.94–1.34) for all leukemia types and any dye users. The current analysis has tighter confidence intervals and included 10 case–control and two cohort studies not considered in the analysis performed by Takkouche.

Smoking is a suggested risk factor for the development of AML, ALL, and CML, potentially due to the presence of benzene, radioactive components, and other carcinogens present in tobacco and tobacco smoke 35, 36, 37, 38. The majority (15/20) of studies in this analysis did not directly control for smoking, and the studies that did control for smoking did not provide risk estimates by leukemia type. When restricted to studies that adjusted for smoking, the magnitude of the risk of leukemia decreased. This provides evidence of potential confounding by smoking if smoking histories differed among users and nonusers of hair dye. For example, an increased prevalence of smoking among hair dye users could result in an increased risk of leukemia, independent of hair dye use, and overestimate the true effect. Because so few studies controlled for smoking, we were unable to perform smoking‐adjusted stratified analyses. This requires further research, as it remains unclear if adjustment for smoking would materially influence the observed outcomes in the stratified analyses.

Data from all studies combined suggest that hair dye use generally does not have a causal effect on the development of leukemia, potentially due to insufficient doses of chemicals present in hair dyes, or the ability of skin cells to detoxify aromatic amines and attenuate systemic exposure 39, 40. Conversely, this crude unstratified meta‐analysis may have diluted certain patterns of use that are associated with an increased risk of developing leukemia. Additionally, a significant amount of heterogeneity was observed in the unstratified model, likely due to the presence of various exposure classifications and study designs. Therefore, this study performed stratified meta‐analyses by various exposure characteristics to understand the potential drivers of risk.

### Risk by study design

When stratified by study type, ever use of hair dye was associated with a statistically significant increased risk of leukemia in case–control studies, which was not observed in cohort studies (Table 2). Hair dye use exposure was generally measured at baseline in cohort studies, which could introduce bias from exposure misclassification if hair dye use behaviors changed during follow‐up. Specifically, nondifferential misclassification of binarily measured exposures would result in a bias in the risk estimate toward the null value, which could explain the observed reduction in the pooled risk estimate among cohort studies. Conversely, case–control studies are susceptible to recall bias if the reporting of exposures is systematically dissimilar between the cases and controls. Among case–control studies, if cases were more likely to report hair dye use exposures, then the pooled risk estimate would be biased away from the null and overestimate the true effect. It is therefore worth noting that (1) only case–control studies reported statistically significant increased risk estimates, and (2) the majority of studies included in the overall and subsequent stratified analyses were case–control studies.

### Risk by hair dye type

Individuals who used dark‐colored hair dye products were at a statistically significant increased risk of leukemia, whereas those that used light‐colored hair dye products were not (Table 2). Dark hair dyes contain a higher concentration of primary aromatic amine intermediates and couplers in order to increase the shade of color 14. Hence, there are more reactive chemical species in dark hair dyes, which could explain the color‐based difference in leukemia risk. Also, users of permanent hair dye were at a statistically significant increased risk of all types of leukemia, which was not observed among semi‐permanent or nonpermanent hair dye users. This result differs from Takkouche 17, potentially due to the increased robustness of the underlying data in the current analysis. Permanent hair dyes undergo an oxidative reaction to impart color to the cortex of the hair shaft, which can result in oxidative damage 4. Oxidative damage has been associated with DNA mutations and cell proliferation during cancer initiation and progression 41. In contrast, semi‐permanent or nonpermanent hair dyes do not penetrate the hair cortex or undergo oxidative reactions, but rather utilize van der Waals forces to adhere to the hair cuticle 4, 15.

### Risk by time period and duration of use

This study also examined time period of hair dye use as a driver of leukemia risk. During the 1970s, the cosmetic industry incorporated aromatic amine ingredients (including 2, 4‐diaminoanisole, 2‐amino‐4‐nitrophenol, and 2, 4‐diaminotoluene) into hair dye formulations that were shown to produce mutagenic activity and dermally penetrate humans, resulting in regulatory action in 1978 to ban certain aromatic amine compounds in the European Union 6, 15, 42, 43, 44, 45. Additionally, in 1980, the Food and Drug Administration (FDA) required the presence of a warning label on hair dyes including 4‐methoxy‐m‐phenylenediamine and 4,‐methyl‐m‐phenylenediamone sulfate, stating that the product “contains an ingredient that can penetrate your skin and has been determined to cause cancer in laboratory animals” 46.

The implication of these time‐varying exposures is that hair dyes prior to reformulation are potentially more carcinogenic than more recent hair dye formulations after the removal of certain mutagenic ingredients. Specifically, this study found that individuals who used hair dye products prior to 1980 were at a statistically significant increased risk of leukemia, which was not observed in individuals that used hair dyes after 1980 (Table 2). All underlying studies were age‐adjusted case–control designs, so these variables likely did not differentially influence the observed outcomes. Among studies that reported “ever” use of hair dye, we were unable to stratify the underlying risk estimates in a systematic way by pre‐ and post‐1980 dye formulations. Therefore, the current observed increased risks among studies may be driven by historical hair dye constituents. However, it should also be noted that the lack of a statistically significant association for more recent post‐1980 hair dye use may be due to insufficient induction and latency periods since first exposure, or reduced cumulative lifetime exposures.

This analysis also provided evidence that duration of use may be a factor in the risk of leukemia, as use of hair dye for ≥15 years was associated with an increased risk of leukemia, while use of hair dye for ≤10 years was associated with a decreased risk of leukemia (Table 2). All underlying studies adjusted for age. However, these findings could also be an artifact of prior formulations, as individuals using hair dyes for ≥15 years may have used hair dye products prior to 1980. Similar to the time period stratification analysis, lack of an increased risk for the ≤10 years group could be due to latency, which has been reported to range from 1.5 to 15 years for leukemia 47.

### Risk by leukemia type and gender

Lymphocytic leukemia develops from lymphocytes while myeloid leukemia arises from primarily granulocytes or monocytes. Similar to Takkouche, we report that hair dye use was associated with an increased risk of lymphocytic leukemia (Table 2). This trend was also observed when further stratified by leukemia subtype, with hair dye use being associated with a higher magnitude of risk for ALL and CLL than AML. We were unable to perform a separate model for CML due to an insufficient number of studies. Research has suggested that lymphocyte cells may be susceptible to hair dye constituents, as it has been noted that DNA damage in lymphocytes was slightly higher in volunteers after hair dying, and that single‐strand DNA breaks were produced in lymphocyte cells exposed to para‐phenylenediamine 48, 49.

Findings also suggest that male users of hair dye are at an increased risk of leukemia, which is an area of research that requires further investigation. This may be due to different hair dye use exposure patterns (i.e., frequency of use, differences in dye types, etc.); however, as there were only four studies that provided male‐specific risk estimates, we were unable to further stratify subanalyses by gender. It should be noted that all studies involving men were case–control studies.

### Strengths and limitations

The main strength of this study is that it provides a pooled estimate of leukemia risk among hair dye users across various study populations. Additionally, the inclusion of more studies allowed for the further investigation of exposure characteristics, including hair dye type and duration/time period of use. A methodological limitation was less accurate exposure classification in studies that assessed the risk of leukemia due to a suite of past occupational and lifestyle exposures, and therefore only collected limited data on the history of lifetime hair dye use 20, 27, 28, 34, 50, 51. Specifically, studies that only collected information on “ever” use of hair dye were unable to adequately characterize exposure profiles that may influence the risk of disease, as shown in the above stratified analyses.

This analysis is also limited by the studied disease classifications, with some studies reporting risk estimates for all leukemia types combined rather than by specific leukemia subtypes. The most informative summary risk measures should be analyzed by leukemia subtype due to different etiologies. However, we were unable to perform meta‐analyses for all leukemia subtypes, and were unable to further stratify subtype meta‐analyses by hair dye type, gender, etc. This body of literature would benefit from additional studies by specific leukemia subtypes. Additionally, the underlying studies did not provide data that enabled us to perform a robust dose–response analysis. While we used duration of use as a proxy of cumulative dose to assess the dose–response relationship, future studies should attempt to characterize different dose levels.

The issue of dependency also arises during the analysis of underlying case–control studies. In studies that calculated risk estimates for non‐Hodgkin lymphoma, multiple myeloma, and leukemia, the same control populations were used in all calculations despite differing disease outcomes. Therefore, these calculations in the underlying studies may not be considered independent, and multiple comparisons may result in observed statistically significant associations that do not exist 52.

## Conclusions

Findings suggest that personal hair dye use is not a significant risk factor for leukemia when data from all studies were combined. Additionally, statistically significant associations were observed when studies were stratified by exposure profile characteristics, including hair dye type, gender, and duration/time period of use. While statistically significant, the clinical significance of these findings remains unclear. Further research is required to determine whether the aforementioned stratified associations truly reflect a risk of leukemia in certain users of hair dye, due to the potential for methodological limitations in the underlying studies (e.g., confounding and recall bias) to overestimate the true effect.

## Conflict of Interest

The authors declare that there are no conflicts of interest.  The authors are employed by Cardno ChemRisk, a consulting firm that provides scientific advice to the government, corporations, law firms, and various scientific/professional organizations. Cardno ChemRisk has not performed consulting work for hair dye manufacturers, and this analysis was designed, conducted, and funded exclusively by Cardno ChemRisk.
